# Outpatient care or less than 48-hour length of hospital stay after open complex abdominal wall surgery: current reality or just a surgeon fantasy?

**DOI:** 10.1097/JS9.0000000000003482

**Published:** 2025-09-06

**Authors:** Gaëtan-Romain Joliat, Eddy Cotte, Guillaume Passot

**Affiliations:** a Department of General Surgery and Surgical Oncology, Centre Hospitalier Universitaire Lyon Sud, Pierre-Bénite, France; b Department of Visceral Surgery, Lausanne University Hospital CHUV, University of Lausanne (UNIL), Lausanne, Switzerland

**Highlights**

- This article discusses tools to achieve an effective recovery after an open complex abdominal wall surgery.
- It is based on the author recent clinical results and current literature.
- During the 2023-2024 period, 94/180 patients (52%) left the hospital within 48 hours after an open complex abdominal wall surgery.
- A total of 68/180 patients (38%) had a <24-hour stay.
- Preoperative optimization, patient information, botulinum toxin injection, intraoperative local anesthesia/block, and enhanced recovery protocols are current tools to obtain the best outcomes after an open abdominal wall surgery.

## Open abdominal wall surgery (AWS) in the current era of minimally-invasive surgery

Worldwide frequency of abdominal hernias has been rising[1], and development of incisional hernias has followed the same trend^[2,3]^. AWS outcomes have an important impact on health care systems, in particular regarding costs and hospital-associated resources[3].

New minimally invasive techniques in laparoscopy or robotic surgery (such as eTEP or posterior component separation) have recently been described and used. Over the past few years, robotic AWS has importantly developed, showing interesting results regarding postoperative recovery, pain, and length of stay (LoS)[4]. However, open AWS remains widely performed and might still be required in several complicated clinical scenarios, where minimally invasive surgery is not feasible.

This research letter discusses the existing tools to achieve effective recovery with short LoS after open complex AWS based on our recent clinical results and current literature. Details on the methods can be found in the supplementary Digital content, materials. Available at: http://links.lww.com/JS9/F86. The present work has been reported in line with the STROCSS criteria[5].

## Results of the presented cohort

From 2021 to 2024, 247 patients underwent open complex AWS in our division (median age 65 years; women 46%; median body mass index 28.7 kg/m^2^). A complex hernia was defined as neck size >10 cm, loss of domain (Sabbagh index >20%), recurrent ventral hernia, or subcostal/parastomal/lateral locations[6].

Seventy-four patients were able to go home within 24 hours after surgery (30%), while 26 patients (11%) were discharged between 24 and 48 hours after surgery. Median LoS was 3 days (IQR 1-5). A significant LoS improvement was noted in the 2023-2024 period (n = 180, median 2 days, IQR 1-5) compared to the 2021-2022 period (n = 67, median 5 days, IQR 3-7, *P* < 0.001). During the 2023-2024 period, 68/180 patients (38%) had <24-hour stay, while 26/180 patients (14%) left the hospital between 24 and 48 hours after surgery compared to 5/67 patients (7%, *P* < 0.001) and 2/67 patients (3%, *P* < 0.001) in the 2021-2022 period.

**Figure 1.**
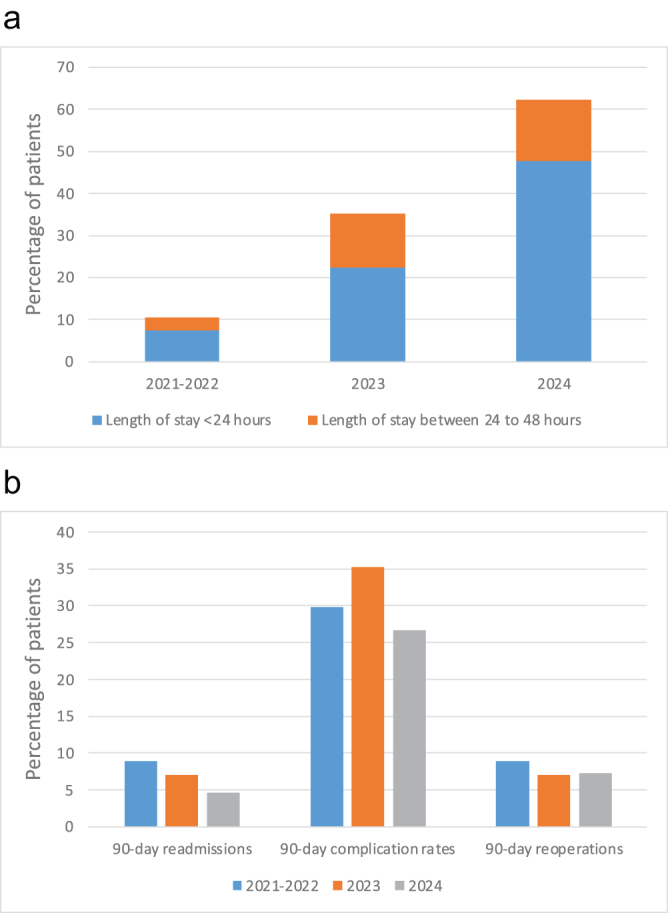
(A) Percentage of patients with lengths of stay <24 hours and between 24 and 48 hours for the 2021-2022, 2023, and 2024 periods (*P* < 0.001). (B) Percentage of 90-day readmissions, 90-day complication rates, and 90-day reoperations for the 2021-2022, 2023, and 2024 periods.

Similar complication rates were found during both periods (2021-2022: 20/67 = 30%, 2023-2024: 54/180 = 30%, *P* = 0.982). Moreover, no increase in the 90-day readmission rates was noted despite LoS decrease (3/67 = 4% vs. 7/180 = 4%, *P* = 0.835). Detailed characteristics and outcomes of overall cohort are depicted in Table 1. Figure 1 illustrates LoS, complication rates, and readmission rates for the 2021-2022, 2023, and 2024 periods. Within a mean follow-up of 16 months (95% CI 15-17), 9 patients presented a recurrence (4%). More details on the recurrence-free survival and predictive factors of LoS ≤ 48 hours are shown in the supplementary Digital content. Available at: http://links.lww.com/JS9/F86

**Table 1:** Characteristics and outcomes of patients (n = 247) who underwent open abdominal wall surgery for complex hernias at our hospital from 2021 to 2024

	Median or number	IQR or percentage
Age, years	65	56-73
Sex (women)	113	46%
BMI, kg/m^2^	28.7	25.4-32.0
ASA score III/IV	62	25%
Smoker	46	19%
Diabetes mellitus	47	19%
Immunosuppression	15	6%
COPD	51	21%
Cirrhosis	3	1%
Prior hernia repair	109	44%
Botulinum toxin injection	81	33%
PPP (Goni-Moreno procedure)	8	3%
Midline hernia	177	72%
Hernia neck size, cm	8	6-10
Extraperitoneal mesh placement	213	86%
Component separation	73	30%
Overall complications	74	30%
Major complications	31	13%
Length of stay, days	3	1-5
Length of stay <24 hours^a^	74	30%
Reoperation within 90 days	19	8%
Readmission	10	4%
Recurrence^b^	9	4%
Reoperation during follow-up	8	3%
Mortality	0	0%
Chronic pain	6	2%
Persistent preoperative symptoms	1	0.4%

BMI: body-mass index, ASA: American Society of Anesthesiologists, COPD: chronic obstructive pulmonary disease, PPP: preoperative progressive pneumoperitoneum, IQR: interquartile range.

^a^ Patients left the hospital within 24 hours of the surgery.

^b^ Mean follow-up was 16 months (95% confidence interval: 15-17).

Even though an experience bias (experience of the surgeon and health-care professional team, organizational improvement, progress of the prehabilitation program over time) cannot be excluded to explain the decrease of LoS in 2023-2024 compared to 2021-2022, it was also at that time point that routine use of epidural analgesia was abandoned. Catheters of epidural analgesia are often kept for 3-5 days, which can prolong LoS. Moreover, prehabilitation was found to be an independent predictor of LoS predictor of LoS ≤48 hours (as shown in the supplementary Digital content), highlighting its importance for an enhanced recovery. Multimodal prehabilitation helps to improve the patient’s general and cardiorespiratory status, to optimize the patient comorbidities, to accompany the patient, and to prepare the patient for a short LoS with active postoperative recovery.

## Existing tools to improve outcomes after open AWS

It is paramount to improve the risk factors of morbidity after open AWS, which can be altered with effective preoptimization. Stringent management of diabetes mellitus, smoking cessation, and weight loss in obese patients improve postoperative morbidity and recurrence rate[7]. Prehabilitation is an important component of patient optimization. In our division, all complex AWS patients were proposed a prehabilitation including nutritional, physical, and psychological support. Patients with caloric-protein deficiencies were given oral nutritional supplements, while specific diets were proposed for obese patients. Physical training included cardiorespiratory exercises and hypopressive physiotherapy. Evidence regarding preoptimization and prehabilitation in AWS remains scant but preliminary data showed clear benefits[8].

For large/complex hernias, preoperative injection of botulinum toxin A (BTA, 4-8 weeks prior to AWS) elongates the lateral abdominal muscles – due to flaccid paralysis by preventing acetylcholine release at the neuromuscular junction – and potentially decreases hernia width[9]. BTA could permit to obtain a closure with less tension, to decrease the need for component separation and of postoperative morbidity; however, these hypotheses remain to be demonstrated. Moreover, BTA could also have a positive impact on postoperative pain and opioid need by reducing the abdominal tension and via a direct chemical anesthetic effect (modulation of substance P release by pain receptors). BTA can be a powerful adjunct for achieving enhanced recovery.

Epidural anesthesia has good analgesic effects postoperatively, but the catheter remains in place for several days, keeping patients in hospital until removal. Other efficient options are intraoperative local infiltration (e.g., retrorectus infiltration) or ultrasound-guided local blocks such as transverse abdominis plane (TAP) or rectus sheath blocks. Epidural avoidance and use of local anesthesia/TAP blocks could permit to reduce LoS without increasing postoperative pain[10]. In our experience, local infiltrations and TAP blocks have been preferably performed instead of epidurals over the last 2 years. Median LoS decreased from 5 to 2 days and <24-hour stays increased from 7% (5/67) to 38% (68/180, *P* < 0.001).

Drain use remains controversial with no clear literature data. While patients can go home with drains, the authors have a no-drain policy for AWS as the authors think that drains do not reduce surgical-site occurrence rates. Absence of drains at home nevertheless eases postoperative care (no dressing change, no need for nurse visit or follow-up appointment for drain removal).

Mesh type, properties, and weight are also important parameters to take into account during the operation. In ventral hernia, synthetic, macroporous meshes have been shown to decrease postoperative complications and long-term recurrence risk[11], while mesh weight does not seem to impact the postoperative course in inguinal and ventral hernia repair^[12,13]^.

During postoperative period, concepts of enhanced recovery should be applied: quick mobilization, early oral nutrition, and avoidance of urinary catheters (or early removal). Enhanced recovery after surgery programs in AWS were shown to decrease LoS[14]. Patients should be prepared preoperatively to know what to expect after surgery (including probable LoS) and be actor in their recovery. Thus, patients are mentally prepared to leave the hospital early and are not surprised once they are discharged.

## Conclusions

Improving postoperative recovery with short LoS and brief off-work period remains a priority in complex AWS. Decreasing LoS has multiple benefits at several levels. At patient level, going home early decreases nosocomial infection risks, improves patient satisfaction (quicker return to their familiar environment stimulating their autonomy and potentially improving the stress associated with an unknown milieu), and facilitates resumption of daily activities with potential positive repercussions on interrupted professional activities. For surgeons and their teams, it can be hypothesized that an enhanced patient recovery could have a positive impact on mental well-being (improvement of psychological load and stress), but this remains to be demonstrated. Finally, short LoS is also beneficial for the health care system in general with decreased societal costs and increased patient turnover.

When considering the significant numbers of AWS performed worldwide, reaching high rates of LoS <48 hours and outpatient operations could lead to substantial savings for hospitals, health care systems, and potentially taxpayers. To put that into perspective, considering that approximately 600,000 ventral hernia repairs/year are performed in the USA with estimated annual costs of $9.7 billion, the potential of pecuniary savings by reducing LoS and improving functional patient recovery after open AWS could be tremendous[15]. To achieve these goals, preoperative optimization with prehabilitation^[16]^, clear patient information of the expected perioperative journey, BTA injection, intraoperative local anesthesia/block, and enhanced recovery protocols are current tools in the surgeon armamentarium to obtain the best outcomes after open AWS.

## Supplementary Material

## Data Availability

The data that support the findings of this study are available from the corresponding author, upon reasonable request.

## Research Registration Unique Identifying Number (UIN)

Not Applicable (Research letter).
