# Supplementary material for: Outpatient care or less than 48-hour length of hospital stay after open complex abdominal wall surgery: current reality or just a surgeon fantasy?
Source: Int J Surg. 2025 Sep 6;112(1):1877–80. doi: 10.1097/JS9.0000000000003482 (PMC12825743; doi:10.1097/JS9.0000000000003482)
Supplement: Supplementary file 1 [file js9-112-1877-001.docx]

**Supplementary materials**

**Methods**

A retrospective cohort study was performed from November 2021 to November 2024.

Prehabilitation for abdominal wall surgery started in October 2021 in our center. Details of the prehabilitation program can be found in a previous publication^16^. Botulinum toxin A (BTA) injection was performed during the entire study period. Indications for BTA injection were hernia defect >8 cm, concomitant large midline hernia and wide diastasis, concomitant lateral and midline hernias, or loss of domain.

Patients were operated on by one surgeon (as primary or supervising surgeon) specialized in abdominal wall surgery. After the operation, patients followed an enhanced recovery program including early mobilization, rapid resumption of oral nutrition, and avoidance or early removal of drains and urinary catheters.

This retrospective article followed the updated STROCSS 2025 guideline^5^.

All patients were candidate for early discharge. Criteria for hospital discharge were pain $\leq$3/10 on a visual analog scale with oral analgesics, food tolerance, and resumption of urination. From 2023, use of epidural analgesia was not routinely used in abdominal wall surgery, and a multimodal pain management strategy with TAP-block, local anesthesia, oral/intravenous medications was preferred. The aim of avoiding epidural analgesia was to potentially decrease length of stay as epidural is often used for 3-5 days.

After discharge, patients were instructed to avoid weightlifting more than 5 kg for 4 weeks. An abdominal binder was given to the patients, and patients were recommended to wear it if they felt more comfortable with it, otherwise they could not use it. No food and exercise restrictions were applied.

A Kaplan-Meier curve was used to illustrate the freedom from recurrence. The reverse Kaplan-Meier method was used to calculate the mean follow-up. A binary logistic regression was performed to find predictive factors of length of stay $\leq$48 hours. Only items with p-value <0.1 on univariable analysis were included in the multivariable regression.

**Results**

The mean recurrence-free survival was 34 months (95% confidence interval: 33-35). The Kaplan-Meier curve for recurrence-free survival is shown hereunder:


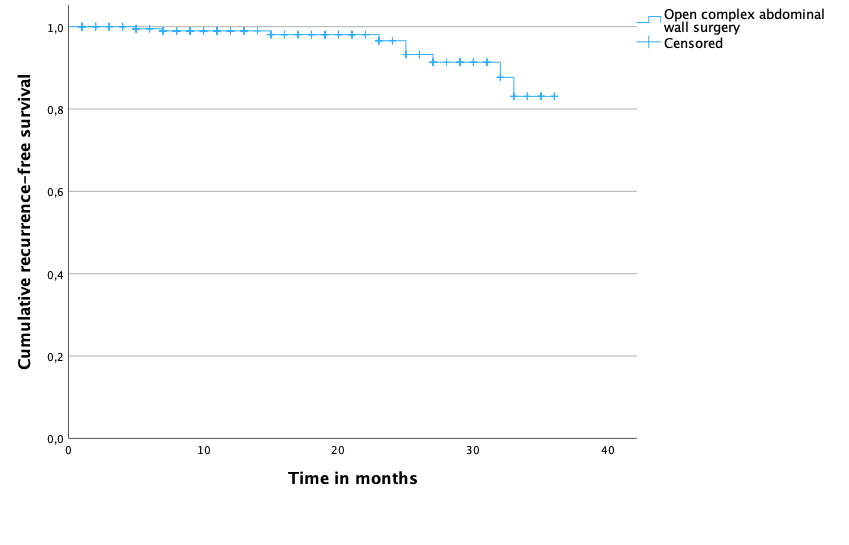


Patients at risk

247 145 81 33 1

The uni- and multivariable binary logistic regression of preoperative predictive factors for length of stay $\leq$48 hours is depicted in the following table:

|  | Univariable, OR (95% CI) | P-value | Multivariable, OR (95% CI) | P-value |
| --- | --- | --- | --- | --- |
| Age, years | 1 (1-1) | 0.827 |  |  |
| Sex, women vs men | 0.8 (0.5-1.3) | 0.269 |  |  |
| ASA score, I/II vs III/IV | 2.2 (1.2-4) | **0.017** | 1.9 (1-3.8) | 0.051 |
| Body mass index, kg/m^2^ | 1 (0.9-1) | 0.309 |  |  |
| Smoking | 1.6 (0.8-3.1) | 0.147 |  |  |
| Diabetes | 0.4 (0.2-0.8) | **0.010** | 0.5 (0.2-1.1) | 0.073 |
| Prehabilitation | 0.3 (0.2-0.5) | **<0.001** | 2.5 (1.3-5) | **0.006** |
| No botulinum toxin use | 0.4 (0.2-0.7) | **0.003** | 0.6 (0.3-1.1) | 0.095 |

ASA: American Society of Anesthesiologists, OR: odds ratio.

**Discussion**

Limitations: The retrospective design of the data collection could have been source of mistakes and biases. Moreover, as mean follow-up was under 2 years, it could be extended in future research to have more long-term data.
